# TractoSCR: a novel supervised contrastive regression framework for prediction of neurocognitive measures using multi-site harmonized diffusion MRI tractography

**DOI:** 10.3389/fnins.2024.1411797

**Published:** 2024-06-26

**Authors:** Tengfei Xue, Fan Zhang, Leo R. Zekelman, Chaoyi Zhang, Yuqian Chen, Suheyla Cetin-Karayumak, Steve Pieper, William M. Wells, Yogesh Rathi, Nikos Makris, Weidong Cai, Lauren J. O'Donnell

**Affiliations:** ^1^Brigham and Women's Hospital, Harvard Medical School, Boston, MA, United States; ^2^School of Computer Science, University of Sydney, Sydney, NSW, Australia; ^3^School of Information and Communication Engineering, University of Electronic Science and Technology of China, Chengdu, China

**Keywords:** diffusion MRI tractography, tractometry, neurocognition prediction, ABCD study, deep learning, contrastive representation learning

## Abstract

Neuroimaging-based prediction of neurocognitive measures is valuable for studying how the brain's structure relates to cognitive function. However, the accuracy of prediction using popular linear regression models is relatively low. We propose a novel deep regression method, namely *TractoSCR*, that allows full supervision for contrastive learning in regression tasks using diffusion MRI tractography. TractoSCR performs supervised contrastive learning by using the absolute difference between continuous regression labels (i.e., neurocognitive scores) to determine positive and negative pairs. We apply TractoSCR to analyze a large-scale dataset including multi-site harmonized diffusion MRI and neurocognitive data from 8,735 participants in the Adolescent Brain Cognitive Development (ABCD) Study. We extract white matter microstructural measures using a fine parcellation of white matter tractography into fiber clusters. Using these measures, we predict three scores related to domains of higher-order cognition (general cognitive ability, executive function, and learning/memory). To identify important fiber clusters for prediction of these neurocognitive scores, we propose a permutation feature importance method for high-dimensional data. We find that TractoSCR obtains significantly higher accuracy of neurocognitive score prediction compared to other state-of-the-art methods. We find that the most predictive fiber clusters are predominantly located within the superficial white matter and projection tracts, particularly the superficial frontal white matter and striato-frontal connections. Overall, our results demonstrate the utility of contrastive representation learning methods for regression, and in particular for improving neuroimaging-based prediction of higher-order cognitive abilities. Our code will be available at: https://github.com/SlicerDMRI/TractoSCR.

## 1 Introduction

The brain's white matter (WM) connections, which can be quantitatively mapped using diffusion MRI (dMRI) tractography (Zhang et al., 2022a), play an important role in brain networks that enable human cognition (Wang et al., 2018; Zekelman et al., 2022). Investigating the predictive relationship between WM microstructure and cognition can therefore improve our understanding of the brain in health and disease. Regression analysis, which can predict values of a dependent variable (label) given a set of input independent variables (features), enables the prediction of neurocognitive measures given input features from neuroimaging. This strategy is recently of high interest (Reddy Raamana and Strother, 2017; Sripada et al., 2020; Chamberland et al., 2021; Kim et al., 2021; Richie-Halford et al., 2021; Feng et al., 2022; Radhakrishnan et al., 2022; Wu et al., 2022). While many studies perform prediction using high-dimensional neuroimaging features from T1-weighted MRI (Aracil-Bolaños et al., 2019; Merz et al., 2022; Weerasekera et al., 2023) or functional MRI (fMRI; Cui and Gong, 2018; Dubois et al., 2018; Sripada et al., 2020; Wu et al., 2022) or multimodal data (Gong et al., 2021, 2022; Kim et al., 2021; Mansour et al., 2021; Radhakrishnan et al., 2022; Sun et al., 2022), a unimodal focus on dMRI tractography (e.g., Jeong et al., 2021; Chen et al., 2022b; Feng et al., 2022; Mansour et al., 2022) can improve our understanding of the role of the WM connections in cognition. While a number of studies have pursued prediction of neurocognitive measures based on information from dMRI tractography, current approaches (Chen et al., 2020a; Jeong et al., 2021; Berger et al., 2022; Zekelman et al., 2022) are limited in terms of study cohorts and regression methodology.

Linear regression models such as ElasticNet (Zou and Hastie, 2005) have been widely used for prediction of neurocognitive performance (Cui and Gong, 2018; Jollans et al., 2019; Li et al., 2020b; Seguin et al., 2020; Sripada et al., 2020; Gong et al., 2021; Madole et al., 2021; Brown et al., 2022; Feng et al., 2022; Jandric et al., 2022; Zekelman et al., 2022), while some studies (Jeong et al., 2021; Chen et al., 2022b; Feng et al., 2022) have explored deep-learning-based regression using multilayer perceptrons (MLP) and convolutional neural networks (CNN). However, the prediction accuracy of linear regression models is relatively low (Sripada et al., 2020), and non-linear regression models may suffer from overfitting, especially on high-dimensional datasets (Cui and Gong, 2018). Developing more advanced methods has the potential to improve prediction accuracy of neurocognitive performance metrics and to provide novel information about specific brain structures that may be important for their prediction.

One avenue for improving the prediction of neurocognitive performance metrics is to investigate recent machine learning algorithms for the analysis of tabular (row and column) data (Borisov et al., 2021). Many quantitative features derived from neuroimaging can be represented as tabular data. The most popular machine learning algorithm for tabular data is the gradient boosting decision tree (GBDT) method (Chen and Guestrin, 2016; Prokhorenkova et al., 2018). In recent years, deep-learning-based methods (Yoon et al., 2020; Arik and Pfister, 2021; Gorishniy et al., 2021; Bahri et al., 2022) have been developed for tabular data, which is the last “unconquered castle” for deep learning (Borisov et al., 2021; Kadra et al., 2021). One important research direction for deep learning on tabular data is representation learning, which can discover beneficial data representations for downstream tasks. For example, the value imputation and mask estimation (VIME; Yoon et al., 2020) and self-supervised contrastive learning using random feature corruption (SCARF; Bahri et al., 2022) methods enable representation learning on tabular data. However, these representation learning methods were developed for classification tasks, and cannot utilize regression label information during representation learning.

Another avenue for improving prediction of neurocognitive measures is to investigate recently proposed algorithms for contrastive learning (Chen et al., 2020b; Khosla et al., 2020; Chen and He, 2021; Sheng et al., 2022). In medical image computing, supervised contrastive learning improves classification accuracy by using labels during representation learning (Dufumier et al., 2021; Schiffer et al., 2021; Zhang et al., 2021; Seyfioğlu et al., 2022; Xue et al., 2023). It is usually designed for classification tasks, where samples with the same categorical label are positive pairs, and samples with different categorical labels are negative pairs. During representation learning, embeddings of positive pairs are pulled together, and embeddings of negative pairs are pushed apart. However, regression tasks require continuous labels (e.g., neurocognitive scores) that cannot directly be used for pair determination. Two recent works have shown that contrastive learning can be useful in the context of regression based on medical images as input (Lei et al., 2021; Dai et al., 2022). For example, RPR-Loc proposed a learning strategy to predict the distance between a pair of image patches (Lei et al., 2021). Recently, the AdaCon method used a contrastive learning strategy that leveraged distances between labels (e.g., bone mineral densities) to benefit downstream computer-aided disease assessment. These recent regression methods did not use labels for pair determination for contrastive learning. How to best use label information to enhance regression is still an open question.

**Figure 1:**
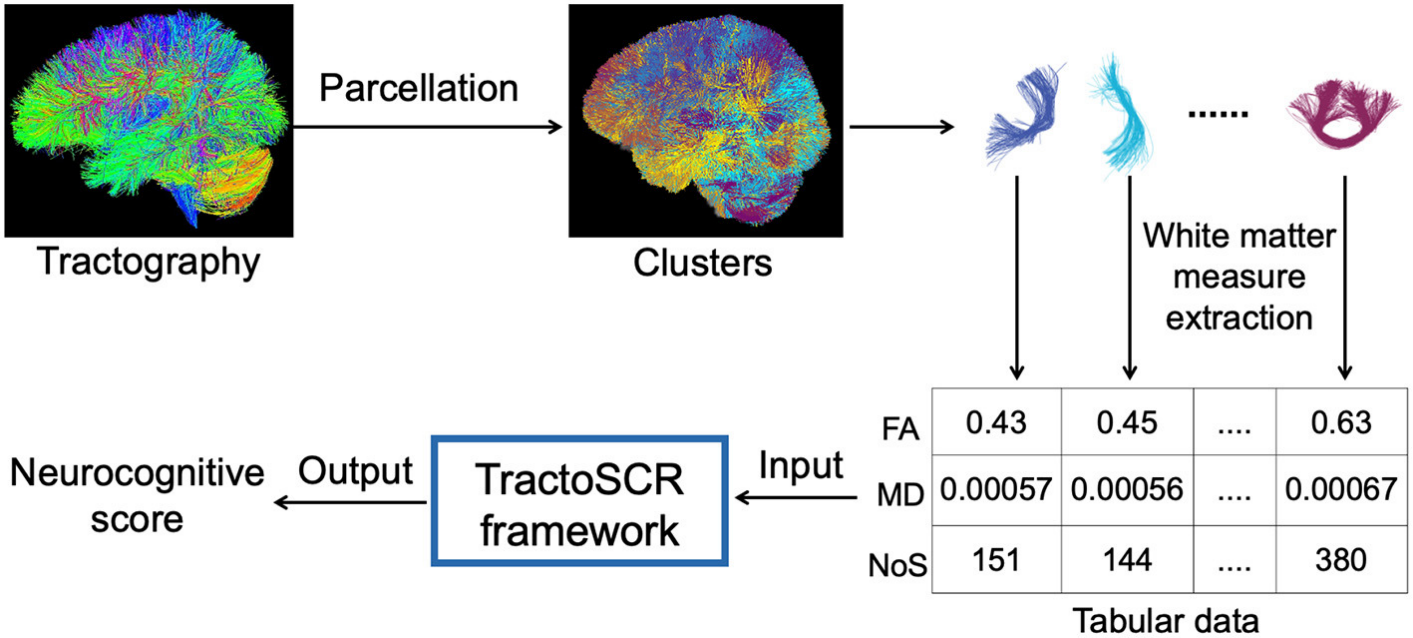
Overview of our proposed TractoSCR framework for neurocognitive score prediction using dMRI tractography. Parcellation of tractography into fiber clusters enables the extraction of cluster-specific white matter measures. These measures are represented as tabular data and input to the TractoSCR framework, which outputs a neurocognitive score. FA, fractional anisotropy; MD, mean diffusivity; NoS, number of streamlines.

In this study, we propose a novel deep regression method for tractography analysis with supervised contrastive regression, referred to as *TractoSCR*. TractoSCR is a novel contrastive representation learning framework to predict measures of neurocognition using white matter microstructure derived from dMRI tractography, as illustrated in Figure 1. Our proposed TractoSCR method extends the supervised contrastive learning method (Khosla et al., 2020), which is designed for categorical data in classification tasks, to perform regression analysis where the predicted labels are continuous values. We propose a novel pair-determination strategy that uses the absolute difference between continuous regression labels to determine positive and negative sample pairs for contrastive learning. To our knowledge, this is the first method that leverages deep representation learning techniques for the prediction of neurocognitive performance. Our method uses a tractography fiber clustering method that enables consistent white matter parcellation across populations. The parcellation allows representation of microstructure features from whole brain tractography as tabular data, which enables the use of a recently proposed random feature corruption technique (Bahri et al., 2022) for data augmentation to further improve prediction performance. In addition, for interpreting prediction results, we propose a novel permutation feature importance algorithm to identify tractography fiber clusters and their corresponding anatomical tracts that are important for prediction of neurocognitive measures. We demonstrate our method in a large-scale dMRI dataset including data from 8735 children, where we explore the relationship between white matter microstructure and prediction of neurocognitive performance (including general ability, executive function, and learning/memory).

The remaining structure of this paper is as follows. Section 2 describes the dataset and data processing, the proposed regression and interpretation methods, and the model training and testing details. Section 3 describes the evaluation metric, experimental results, and interpretation of results. Finally, the discussion and conclusion are given in Sections 4 and 5, respectively.

## 2 Materials and methods

### 2.1 ABCD dataset, tractography parcellation, and microstructural measures

This study includes dMRI data and neurocognitive component scores from the Adolescent Brain Cognitive Development (ABCD) dataset for 8,735 American children (4,560 males and 4,175 females) between the ages of 9–11 (9.9 ± 0.6) across 21 data collection sites (Casey et al., 2018; Volkow et al., 2018; Download at: https://nda.nih.gov/abcd). Three neurocognitive principal component scores from ABCD were studied, representing three major domains of higher-order cognition, namely *General Ability* (PC1), *Executive Function* (PC2), and *Learning/Memory* (PC3; Thompson et al., 2019). These component scores are lower dimensional representations of nine assessment measures from the ABCD neurocognitive battery (Luciana et al., 2018) [including seven measures from the NIH toolbox (Casaletto et al., 2015)]. These component scores statistically summarize nine neurocognitive assessment measures and reveal latent variables which have been theorized to be a more pure reflection of the cognitive domains of interest (Snyder et al., 2015; Thompson et al., 2019). Furthermore, these component scores have been associated with measures of psychopathological behavior (i.e., stress reactivity and/or externalizing behaviors), perhaps suggesting their clinical utility (Thompson et al., 2019).

The ABCD dMRI data was harmonized (Cetin Karayumak et al., 2019; Cetin-Karayumak et al., 2021, 2022; Zhang et al., 2022b) to remove scanner-specific biases, allowing for a large-scale data-driven way to study relationships between brain microstructure and neurocognition. The dMRI harmonization method (Cetin Karayumak et al., 2019) retrospectively removes scanner-specific differences from raw dMRI signals across disparate sites and acquisition parameters, while preserving inter-subject biological variability (e.g., fractional anisotropy (FA) values; Zhang et al., 2022b).

A two-tensor Unscented Kalman Filter (UKF) tractography method (Malcolm et al., 2010; Reddy and Rathi, 2016) was conducted on harmonized dMRI data of all subjects to obtain whole-brain tractography (https://github.com/pnlbwh/ukftractography). The UKF method fitted a mixture model of two tensors to the diffusion data while tracking streamlines. This enabled the estimation of fiber-specific microstructural measures from the first tensor, which models the tract being traced (Reddy and Rathi, 2016). Next, automated parcellation of tractography was performed based on an anatomically curated cluster atlas (Zhang et al., 2018; https://github.com/SlicerDMRI/ORG-Atlases), which was provided by the O'Donnell Research Group (ORG). Compared to traditional tractography parcellation based on cortical atlases, this clustering method was shown to be more reproducible and consistent across the lifespan (Zhang et al., 2018, 2019). For each subject, the ORG atlas (Zhang et al., 2018) enabled extraction of 953 expert-curated fiber clusters. These finely parcellated fiber clusters are grouped and categorized into 58 deep white matter tracts including major long range association and projection tracts, commissural tracts, and tracts related to the brainstem and cerebellar connections, as well as 198 short and medium range superficial fiber clusters. We performed tractography quality control and white matter parcellation using open-source WhiteMatterAnalysis software (https://github.com/SlicerDMRI/whitematteranalysis). Tractography visualization was performed using SlicerDMRI software (dmri.slicer.org; Norton et al., 2017; Zhang et al., 2020).

For all subjects, cluster-specific microstructural measures of fractional anisotropy (FA), mean diffusivity (MD), and number of streamlines (NoS) were computed. These measures have been previously shown to be associated with neurocognitive scores (Madole et al., 2021; Chen et al., 2022c; Zekelman et al., 2022). Here, FA and MD are measures of fiber-specific tissue microstructure, while NoS is widely used to quantify the connectivity strength (Zhang et al., 2022a). These cluster-specific measures can be considered as tabular data, allowing algorithms from the field of tabular data to be employed. For any empty cluster (due to variability of tractography or the underlying anatomy), each measure was set to zero, as in He et al. (2022).

### 2.2 Supervised contrastive regression

**Figure 2:**
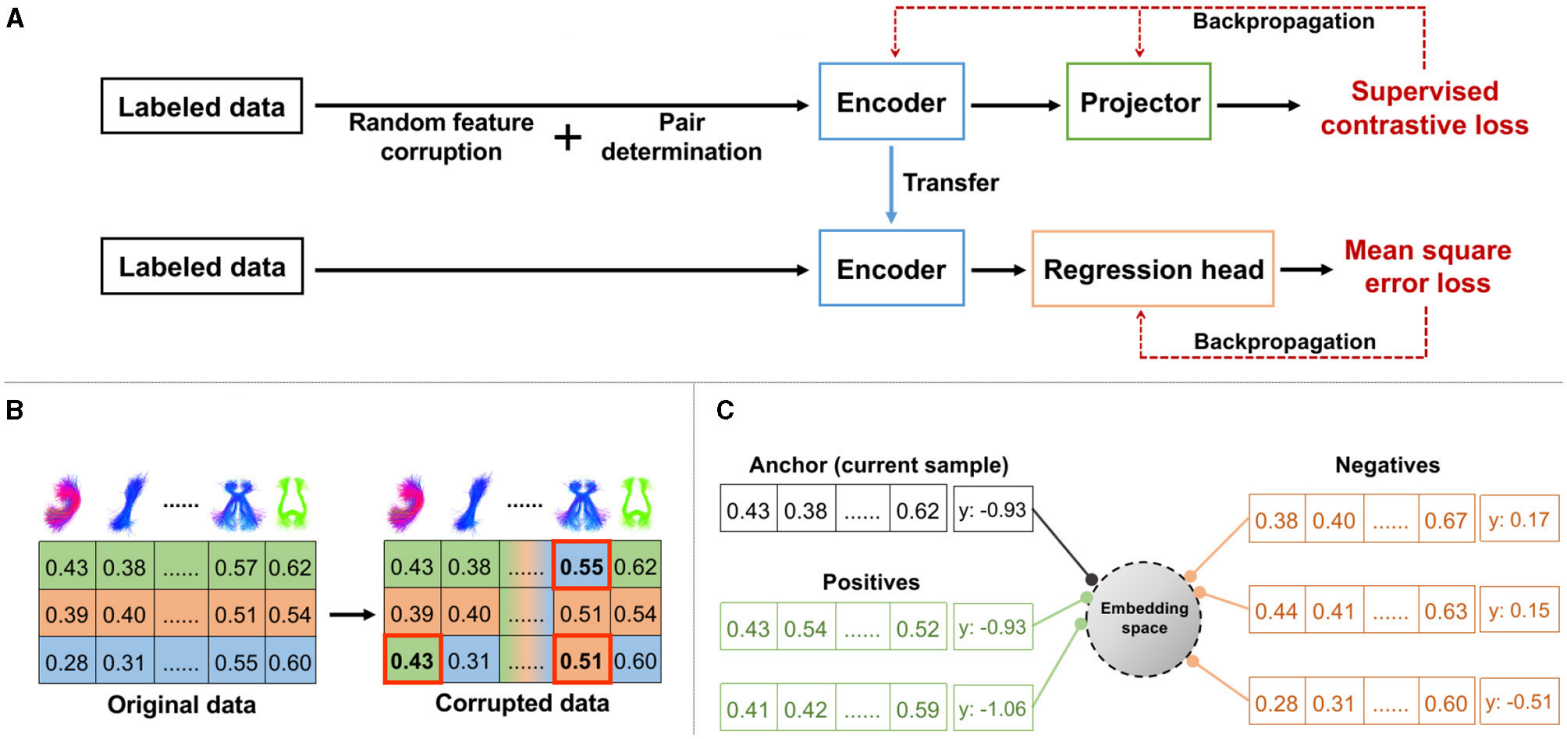
TractoSCR framework: **(A)** overview of contrastive representation learning and fine-tuning, **(B)** random feature corruption for data augmentation with a measure of interest (e.g., FA; rows are randomly selected samples, and columns are cluster-specific microstructural measures), **(C)** positive and negative pairs determination with regression labels (e.g., PC1).

We propose a novel contrastive representation learning method for regression, *TractoSCR*. Our overall strategy is to use the absolute difference between two continuous regression labels to determine positive and negative pairs for contrastive learning. An overview of the TractoSCR framework is shown in Figure 2. The regression framework (Figure 2A) has two phases: contrastive representation learning and fine-tuning. In representation learning, random feature corruption (Figure 2B) and proposed pair determination (Figure 2C) are utilized with a supervised contrastive loss. The network trained in representation learning is then fine-tuned to output neurocognitive scores. These steps are described in the following sections.

#### 2.2.1 Random feature corruption for data augmentation

To avoid potential model overfitting and increase the discriminative ability of the learned global features in contrastive learning, we performed a data augmentation process to create more training samples. We applied the recently proposed random feature corruption technique that was designed specifically for tabular data (Yoon et al., 2020; Bahri et al., 2022). In brief, in each mini-batch of training with input samples *X*, we created a corrupted batch copy $\tilde{X}$. To do so, we chose a proportion of the input cluster-specific measures (features) uniformly at random and replaced each of those measures by a random draw from the corresponding measure dimension of other samples (as shown in Figure 2B). The ratio of replaced measures to all measures is defined as the corruption rate *c*. Corrupted samples $\tilde{X}$ retain the same regression labels *Y* as original samples *X*.

#### 2.2.2 Positive and negative pairs determination

From the generated augmented data in each training mini-batch, we construct positive and negative sample pairs to enable supervised contrastive learning (SCL). Unlike existing studies (Khosla et al., 2020) using SCL to perform a classification task, where positive and negative pairs are defined based on the class labels, determination of positive and negative sample pairs is not straightforward in regression because the regression labels are continuous values. To handle this, we propose a new strategy that uses the absolute difference between two continuous regression labels to determine pairs (Figure 2C). Given *x*_*i*_, *x*_*j*_∈*X* with labels *y*_*i*_ and *y*_*j*_, if |*y*_*i*_−*y*_*j*_| < θ, *x*_*i*_ and *x*_*j*_ are defined as positive pairs. Otherwise, *x*_*i*_ and *x*_*j*_ are considered to be negative pairs. The label difference threshold θ, a threshold on the absolute difference of two regression labels, is the key parameter for positive and negative pair determination. For our dataset with regression labels ranging from ~–3 to 3, the optimal θ is 0.35 based on experimental results. Note that our TractoSCR method is robust to changes in this threshold (from 0.1 to 0.5) as described in Section 3.2.4.

#### 2.2.3 Supervised contrastive loss

After positive and negative pairs are determined using regression labels, the supervised contrastive loss as shown below becomes applicable:


$$L=\underset{r\in R}{\sum }L_{r}=\underset{r\in R}{\sum }\frac{-1}{\left|P\left(r\right)\right|}\underset{p\in P\left(r\right)}{\sum }\log\frac{\exp\left(z_{r}\cdot z_{p}/\tau \right)}{\underset{a\in A\left(r\right)}{\sum }\exp\left(z_{r}\cdot z_{a}/\tau \right)},$$

where *r* is the anchor (current) sample, and *R* is the set of all samples (*X* and $\tilde{X}$) in a training batch (*r* ∈ *R*); *P*(*r*) is the set of samples that are positive pairs with anchor sample *r* (*p* ∈ *P*(*r*)); *A*(*r*) is the set of all samples in *R* except for anchor sample *r* (*a* ∈ *A*(*r*) ≡ *R*\{*r*}); *z*_*r*_, *z*_*p*_ and *z*_*a*_ are contrastive features obtained from *Proj*(·) for samples *r*, *p* and *a*; and τ (temperature) is a tuneable hyperparameter for the contrastive loss.

#### 2.2.4 Contrastive learning and fine-tuning

The overall process of contrastive learning and fine-tuning (Figure 2A) is as follows. In contrastive representation learning, training samples (from *X* and $\tilde{X}$) are input into the encoder *Enc*(·) and projector *Proj*(·) to get embeddings (*Z* and $\tilde{Z}$). The supervised contrastive loss is computed using normalized embeddings (*Z* and $\tilde{Z}$), where positive and negative pairs are determined by absolute differences between regression labels *Y*. After the contrastive representation learning, the parameters of *Enc*(·) are frozen and the *Proj*(·) is untouched, as in Chen et al. (2020b), Khosla et al. (2020), Bahri et al. (2022), and Xue et al. (2022). The usage of *Proj*(·) may retain useful information for downstream regression tasks in *Enc*(·) (Chen et al., 2020b). A predictor head for regression *Reg*(·) is added on top of the trained *Enc*(·). *Reg*(·) takes the output of *Enc*(·) as the input and is fine-tuned with MSE loss to obtain the final prediction.

### 2.3 Ensemble learning

We use ensemble learning (Hastie et al., 2009) to combine prediction results from three predictors that are trained on three microstructural measures (FA, MD, and NoS) independently, as in He et al. (2022). The ensemble prediction is obtained as the average prediction across the three predictors. Therefore, ensemble learning is beneficial in our application to study the relationship between three microstructural measures and neurocognitive performance metrics. Ensemble learning can also potentially improve the performance of the regression, because different microstructural measures may provide complementary information for prediction of neurocognitive performance (Note that ensemble learning is used not only for our method but also for all compared methods in experiments).

### 2.4 Permutation feature importance

We propose a permutation feature importance algorithm to assess the contribution of each cluster to the prediction of a neurocognitive score. Our proposed interpretation method is based on the permutation feature importance (Breiman, 2001), which is a popular model-agnostic technique for estimating how important a feature is for a particular model. The traditional permutation feature importance is defined as the decrease in a model score (e.g., prediction accuracy) when a single feature value is randomly shuffled (permuted) across samples. This enables identification of highly important features that have a large effect on the model's prediction accuracy. This traditional permutation feature importance method is not directly applicable to our high-dimensional data because the decrease of prediction accuracy is negligible when only permuting a single feature value (our input includes 953 cluster-specific white matter features per subject). Therefore, we propose a new strategy to permute multiple feature values simultaneously (e.g., a random sample of 10% of features). By repeating this strategy a very large number of times (e.g., 50,000), we can estimate the importance of all high-dimensional input features.

### 2.5 Implementation details

For model training and performance evaluation, datasets are split into train/validation/test with the rate 70/10/20%, and we repeat each experiment 10 times with different train/validation/test splits to report the average performance. Regarding the network structure, as suggested in Bahri et al. (2022), *Enc*(·), *Proj*(·), and *Reg*(·) all have hidden dimension 256 with the ReLU activation in each layer. *Enc*(·) has four layers, whereas *Proj*(·) and *Reg*(·) both consist of two layers. For training hyperparameters, all deep learning methods are trained with the Adam optimizer with the learning rate 0.001 and use early stopping with patience 3 on the validation loss as in Bahri et al. (2022). We conduct a grid search for parameter selection with *b*∈{256, 512, 1, 024, 2, 048, 4, 096}, *c*∈{0.3, 0.4, 0.5, 0.6, 0.7}, and τ∈{0.5, 1, 5, 10} for our method and all compared representation learning methods. For AdaCon, we also tune the temperature scaling factor (*s*∈{10, 50, 100, 150}) based on their paper and code. Weight ratios of two losses in AdaCon are tuned with the rule that two losses should have similar values (Dai et al., 2022). Then we choose batch size *b* of 2,048, corruption rate *c* of 0.5, and temperature τ of 1 for our contrastive representation learning. Note that our method is not sensitive to hyperparameter changes and has good performance overall. Results with other parameter settings are presented in Section 3.2.4 to demonstrate the robustness. A typical batch size of 128 is chosen in fine-tuning for all deep learning methods. Experiments are performed with Pytorch [16] (v1.8) on a NVIDIA GeForce RTX 2080 Ti GPU machine. For TractoSCR, each experiment (including training, validating, and testing) takes about 30 s with 1.67 GB GPU memory usage.

For the interpretation of prediction results, we implement our proposed feature permutation algorithm for prediction of three neurocognitive measures (PC1, General Ability; PC2, Executive Function; PC3, Learning/ Memory) independently. For each permutation, we shuffle 95 out of 953 feature values across samples in the training dataset. Then we train using TractoSCR. The prediction accuracy is evaluated on the testing dataset, and the decrease of prediction accuracy (compared to the original prediction accuracy) is recorded along with the indices of the 95 shuffled features. For each of the 10 train/validation/test data distributions, we repeat this experiment 50,000 times (50,000 permutations). We obtain final overall importance scores for each feature (cluster) by averaging all recorded decreases of prediction accuracy from all permutations of that feature. Finally, three importance scores are obtained for each cluster, corresponding to the three prediction tasks.

## 3 Results

### 3.1 Evaluation metric

We computed Pearson correlation coefficients (Pearson's *r*) between the ground truth scores and predicted scores to quantify the prediction accuracy. The Pearson correlation coefficient is widely used for evaluation of cognitive prediction from neuroimaging data (Cui and Gong, 2018; Jollans et al., 2019; Sripada et al., 2020; Gong et al., 2021; Mansour et al., 2021; Chen et al., 2022c; Feng et al., 2022; Jandric et al., 2022). It measures the linear correlation (normalized cosine similarity) between two sets of data. A higher value of *r* indicates a better prediction accuracy. We repeated each experiment 10 times with different train/validation/test splits (all methods use the same split). The mean and standard deviation of Pearson correlation coefficients across 10 splits are reported. To evaluate if differences of Pearson's *r* values (10 splits) between our method and compared methods are significant, we implemented a repeated measure ANOVA test for all methods, and then we performed multiple paired Student's *t*-tests between our method and each compared method.

### 3.2 Evaluation results

#### 3.2.1 Comparison of representation learning methods

We compared our proposed TractoSCR with one classical method (AutoEncoder; Rumelhart et al., 1986), two recently proposed methods (VIME; Yoon et al., 2020, and SCARF; Bahri et al., 2022) for representation learning using tabular data, and one recent contrastive learning method (AdaCon; Dai et al., 2022) for medical image-based regression. The autoencoder method is widely used for learning efficient representations. Here, the autoencoder has the same input as TractoSCR and the output has the same dimensionality as the input, and the MSE loss is applied. VIME uses a novel pretext task and data augmentation method for representation learning, and SCARF uses contrastive learning with random feature corruption. AdaCon utilizes its proposed contrastive loss together with an MSE loss for training, and for fair comparison to our method, we apply random corruption for data augmentation for AdaCon. In our study, we train these methods using the suggested settings in their papers and released codes.

Table 1 shows that our proposed method outperforms all compared representation learning methods on the three prediction tasks. The improvements between our method and compared methods (except AdaCon on PC2) are shown to be significant by paired Student's *t*-tests. In addition, our method and AdaCon perform better than other representation learning methods. This result demonstrates the effectiveness of utilizing the relationship between regression labels during contrastive learning. Furthermore, compared to AdaCon, the prediction accuracy of our method achieves relative improvements of 2.4, 2.6, and 6.7% on the prediction of three neurocognitive measures. This illustrates that using regression labels to enable positive and negative pair determination in contrastive learning can improve results on prediction of neurocognitive measures.

**Table 1:** Comparison results (mean and standard deviation of Pearson's *r* across splits) for prediction of three neurocognitive component scores, PC1 (general ability), PC2 (executive function), and PC3 (learning/memory).

	**Methods**	**PC1**	**PC2**	**PC3**
Representation learning comparison	Autoencoder	0.406 ± 0.016 ^*^^*^^*^	0.217 ± 0.022 ^*^^*^^*^	0.234 ± 0.021 ^*^^*^^*^
	VIME	0.407 ± 0.013 ^*^^*^^*^	0.218 ± 0.014 ^*^^*^	0.235 ± 0.017 ^*^^*^^*^
	SCARF	0.411 ± 0.013 ^*^^*^^*^	0.217 ± 0.019 ^*^^*^	0.239 ± 0.020 ^*^^*^^*^
	AdaCon	0.414 ± 0.013 ^*^	0.235 ± 0.021 n.s.	0.253 ± 0.020 ^*^^*^
SOTA regression models	ElasticNet	0.400 ± 0.018 ^*^^*^^*^	0.206 ± 0.021 ^*^^*^^*^	0.237 ± 0.016 ^*^^*^^*^
	GBDT	0.390 ± 0.012 ^*^^*^^*^	0.219 ± 0.019 ^*^	0.238 ± 0.021 ^*^^*^^*^
	MLP (baseline)	0.409 ± 0.018 ^*^^*^^*^	0.209 ± 0.020 ^*^^*^^*^	0.236 ± 0.020 ^*^^*^^*^
Ablation study	TractoSCR*_no − pd − fc_*	0.407 ± 0.016	0.210 ± 0.019	0.231 ± 0.013
	TractoSCR*_no − fc_*	0.419 ± 0.011	0.232 ± 0.020	0.256 ± 0.013
TractoSCR (ours)		**0.424** **±0.014**	**0.241** **±0.014**	**0.270** **±0.015**

The ANOVA test obtains very low *p*-values (1 × 10^−6^ — 1 × 10^−11^). Asterisks show that the difference between our proposed method and the compared method is significant using a paired Student's *t*-test (^*^p < 0.05, ^*^^*^p < 0.01, ^*^^*^^*^p < 0.001, and n.s. is not significant).

#### 3.2.2 Comparison of state-of-the-art methods for regression

We also compared our proposed method with two SOTA machine learning methods for regression (ElasticNet; Zou and Hastie, 2005 and GBDT; Chen and Guestrin, 2016; Prokhorenkova et al., 2018). ElasticNet is popularly used in cognitive prediction (Cui and Gong, 2018; Gong et al., 2021). It performs linear regression with L1 and L2 regularization. We used the implementation in the sklearn package (Pedregosa et al., 2011). GBDT is a strong non-deep competitor for deep learning methods in tabular data (Gorishniy et al., 2021). It iteratively constructs an ensemble of weak decision tree learners through boosting. We selected XGBoost (Chen and Guestrin, 2016), one of the most popular implementations of GBDT, for comparison. Parameters were tuned based on suggestions in Gorishniy et al. (2021). In addition to the above SOTA methods, we also included a multilayer perceptron (MLP) that has the same network structure as ours for a baseline comparison. As shown in Table 1, MLP (our baseline) outperforms ElasticNet and is competitive with GBDT. These results illustrate the power of deep learning methods for neurocognitive score prediction. In addition, compared to the MLP baseline, our proposed method obtains relative improvements in prediction accuracy of 3.7, 15.3, and 14.4% on all three prediction tasks. The improvement between our method and the MLP baseline is very significant (*p* < 0.001) by paired Student's *t*-tests. This demonstrates the effectiveness of our proposed TractoSCR method.

#### 3.2.3 Comparison of ablated versions

An ablation study was conducted with two ablated versions (TractoSCR_*no-pd-fc*_ and TractoSCR_*no-fc*_) of our proposed approach. TractoSCR_*no-pd-fc*_ performs contrastive learning without using regression labels for pair determination and without using random feature corruption. TractoSCR_*no-fc*_ uses regression labels for pair determination but does not perform random feature corruption. As shown in Table 1, the comparison between TractoSCR_*no-pd-fc*_ and TractoSCR_*no-fc*_ illustrates a large improvement when using regression labels for pair determination in contrastive learning. In addition, by applying random feature corruption for data augmentation, the performance improves on all tasks.

#### 3.2.4 Experiments under different hyperparameter settings

**Figure 3:**
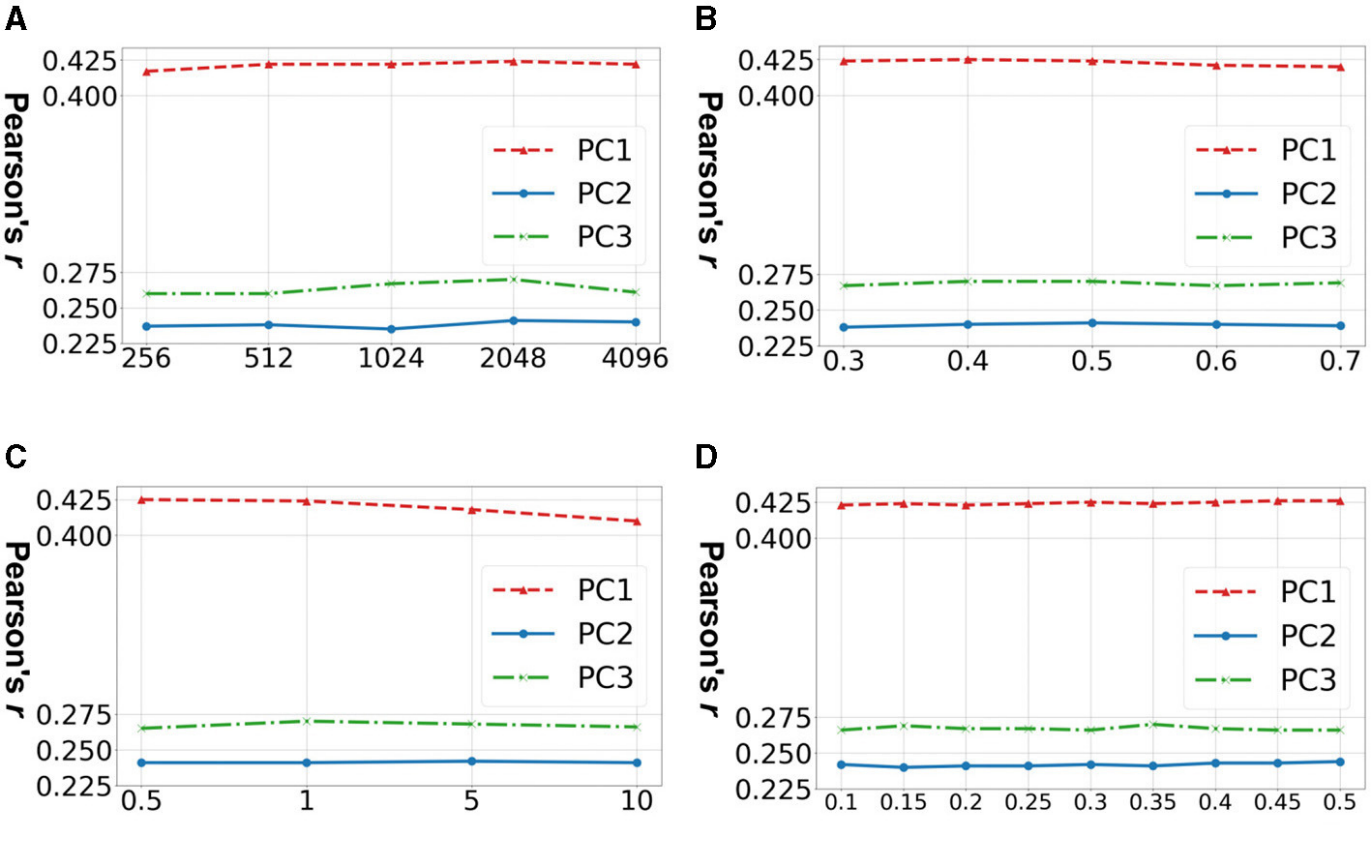
Hyperparameter sensitivity experiments for TractoSCR. Results (Pearson's *r*) on predicting three neurocognitive component scores (PC1, PC2, and PC3) across different hyperparameters: **(A)** batch size *b*, **(B)** corruption rate *c*, **(C)** temperature τ, and **(D)** label difference threshold *θ*. Results demonstrate that TractoSCR is *hyperparameter-insensitive*.

Figure 3 shows the accuracy of prediction of three neurocognitive component scores across four important hyperparameters in TractoSCR. Overall, TractoSCR achieves consistently high prediction accuracy (Pearson's *r*) on all three tasks, which demonstrates TractoSCR is robust to hyperparameter change. Batch sizes and temperatures are important to contrastive learning frameworks in general (Chen et al., 2020b; Khosla et al., 2020). Figures 3A, C show that TractoSCR obtains similar results when the batch size changes from 256 to 4,096 and the temperature changes from 0.5 to 10. Corruption rates control how heavy the data augmentation is in contrastive learning (Yoon et al., 2020; Bahri et al., 2022). As shown in Figure 3B, a negligible change of the result occurs when corruption rates are varied from 0.3 to 0.7. The label difference threshold *θ* is the key parameter for positive and negative pair determination in TractoSCR. As shown in Figure 3D, TractoSCR performs well under different θ thresholds ranging from 0.1 to 0.5.

### 3.3 Interpretation results

**Figure 4:**
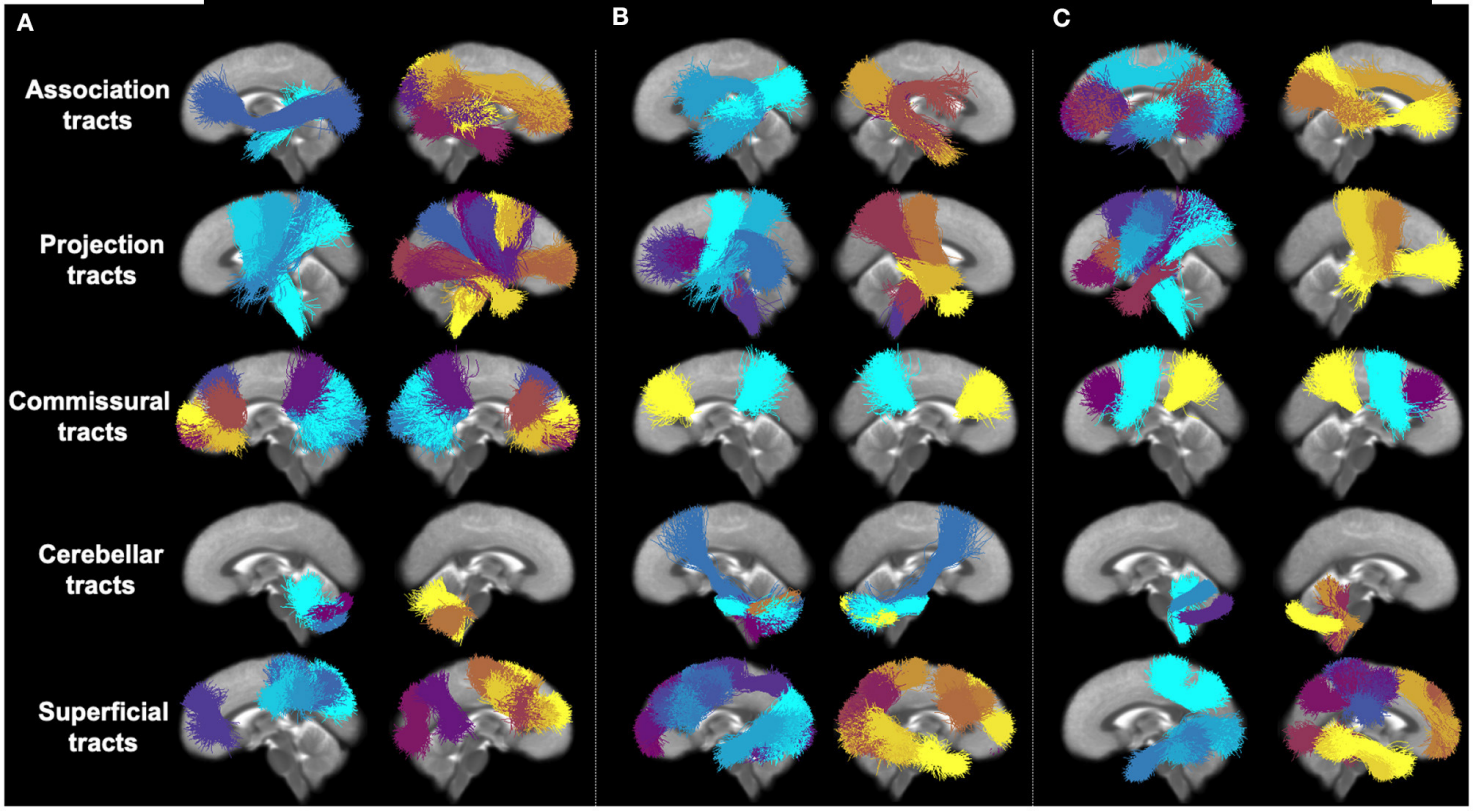
Visual presentation of most predictive fiber clusters (with the 50 highest importance scores) for each individual prediction task. Different fiber clusters are depicted in different colors and organized according to five anatomical tract categories. **(A)** PC1 (general ability). **(B)** PC2 (executive function). **(C)** PC3 (learning/memory).

Figure 4 provides a visualization of the most predictive fiber clusters (defined as the fiber clusters with the top 50 highest importance scores for each prediction task). Together, these fiber clusters may form part of the putative structural networks relating to general cognitive ability (PC1), executive function (PC2), and learning/memory (PC3). The predictive fiber clusters span across all five anatomical tract categories (association, cerebellar, commissural, projection, and superficial tracts; Zhang et al., 2018) and are found in both the left and right hemispheres. This finding is in line with neurocognitive research demonstrating that higher order cognitive functions, such as the ones presently under investigation, are broadly distributed across the brain (Goddings et al., 2021). When this result is examined in detail, we find that the predictive fiber clusters are predominantly located within the superficial and projection white matter (Table 2). This finding contrasts with the relative plethora of white matter and cognition studies that have focused on the role of the association connections (e.g., language in arcuate fasciculus, memory in the uncinate fasciculus, etc.; Forkel et al., 2022). Details about the location of all predictive fiber clusters (Figure 4) within specific tracts (as defined in the anatomically curated ORG atlas, Zhang et al., 2018) are provided in Supplementary Table 1. In addition, we also ran our proposed feature permutation algorithm across the five representation methods (Autoencoder, VIME, SCARF, AdaCon, and TractoSCR) shown in Table 1. These methods' most predictive fiber clusters have a 28–34% overlap for PC1, PC2, and PC3 neurocognition prediction tasks (28% for PC1, 30% for PC2, and 34% for PC3). This result demonstrates the robustness of interpretation in terms of which fiber clusters are most predictive. Overall, the most predictive tracts are the superficial frontal white matter and striato-frontal connections, which have the highest number of clusters found to be important across the three prediction tasks.

**Table 2:** Number of predictive fiber clusters within each anatomical category.

	**PC1**	**PC2**	**PC3**
Association	8 (16.0%)	7 (14.0%)	13 (26.0%)
Projection	**15 (30.0%)**	9 (18.0%)	**14 (28.0%)**
Commissural	10 (20.0%)	2 (4.0%)	3 (6.0%)
Cerebellar	5 (10.0%)	5 (10.0%)	6 (12.0%)
Superficial	12 (24.0%)	**27 (54.0%)**	**14 (28.0%)**
Total	50 (100.0%)	50 (100.0%)	50 (100.0%)

Categories with the highest number of predictive clusters are in bold.

## 4 Discussion

In this study, we proposed a novel deep-learning-based regression method that enables improved prediction accuracy of neurocognitive measures. To our knowledge, we are the first to focus on deep representation learning for neuroimage-based prediction of neurocognitive measures. Unlike commonly used regression methods (Li et al., 2020b; Madole et al., 2021; Brown et al., 2022; Feng et al., 2022), the proposed TractoSCR method allows us to effectively leverage information from regression labels during contrastive learning. A new strategy was proposed to use the absolute difference between two continuous regression labels to determine positive and negative pairs. We also employed random feature corruption, a data augmentation method for tabular data, in contrastive learning. By applying random feature corruption, the performance improved on all prediction tasks (e.g., a relative improvement of 5.5% on PC3).

Our proposed method achieved significantly better prediction performance on a large-scale ABCD dataset in comparison with existing methods, including SOTA regression methods and representation learning methods. For example, on PC3, our method outperformed the SOTA contrastive learning method (AdaCon) with a relative improvement of 6.7% in Pearson's *r*, and our method outperformed the baseline method (MLP) with a relative improvement of 14.4% in Pearson's *r*. We also illustrated that TractoSCR is robust to changes of hyperparameters (batch size *b*, corruption rate *c*, temperature τ, and label difference threshold θ). These results demonstrate the utility of contrastive representation learning methods for the neuroimaging-based prediction of higher-order cognitive abilities. In this study, we obtained Pearson's *r* values ranging from 0.24 to 0.43, indicating a moderate correlation between investigated white matter microstructural measures and neurocognitive scores. Our moderate correlation finding is in general in line with a body of recent work that uses neuroimaging measures to predict cognition (Sripada et al., 2020; Gong et al., 2021; Kim et al., 2021; Feng et al., 2022).

Predicting neurocognitive measures from the ABCD dataset is an interesting but challenging task that has been undertaken using various MRI modalities (Pohl et al., 2019; Sripada et al., 2020; Ooi et al., 2022). For example, T1-weighted MRI was used to predict fluid intelligence scores (Pohl et al., 2019), while a comparison across modalities suggested that information from fMRI could best predict a summary cognition score derived from 36 behavioral scores (Ooi et al., 2022). One recent study by Sripada et al. (2020) used resting-state fMRI to predict the same neurocognitive component scores (PC1, PC2, and PC3) that we have investigated in the current study. Their method obtained Pearson's *r* values of 0.33, 0.09, and 0.15 for the prediction of PC1, PC2, and PC3, respectively (Sripada et al., 2020). These results were based on a smaller dataset (2,013 subjects from the first ABCD data release) and are not directly comparable to our results. However, we note that using tractography fiber cluster microstructure features as input and our novel TractoSCR regression framework for prediction, we obtained higher Pearson's *r* coefficients of 0.42, 0.24, and 0.27 for the prediction of PC1, PC2, and PC3, respectively. As an additional experiment, we also included an additional two measures (tensor 2 FA and MD), which improved the performance by 2.1, 9.1, and 3.7% to give Pearson's *r* coefficients of 0.43, 0.26, and 0.28 for PC1, PC2, and PC3 prediction tasks, respectively. Tensor 2 FA and MD are diffusion measures derived from the second diffusion tensor (representing crossing fibers) using the UKF tractography method. This additional experiment shows that adding more diffusion measures can further improve the performance of neurocognition prediction. Overall, this suggests that fiber cluster measures can potentially provide highly informative features, in combination with TractoSCR that achieves higher prediction accuracy than commonly used linear regression methods.

In our data-driven analysis of imaging and neurocognitive data from 8,735 participants of the ABCD study, we found that fiber clusters within the projection and superficial white matter were the most important for predicting neurocognitive scores related to general cognitive ability, executive function, and learning/memory. This result was enabled by the proposed permutation feature importance algorithm for identifying predictive features from high-dimensional input. This finding may highlight the need for more investigations of the superficial and projection pathways in the context of cognition.

Potential limitations and future work of the present study are as follows. First, in the present study, we explored the relationships between neurocognitive scores and fiber cluster microstructural measures from a single imaging modality, dMRI. Future work may investigate TractoSCR for predicting neurocognitive scores based on features from multiple MRI modalities. Second, we focused on prediction of neurocognitive scores in healthy children. Future work may investigate the proposed TractoSCR framework to predict cognition in the context of aging or disease (e.g., Alzheimer's Disease; Fisher et al., 2019). Third, we employed a relatively simple MLP network. Future developments can include the incorporation of more advanced deep learning networks (e.g., transformer; Vaswani et al., 2017) and recently proposed regression losses (Engilberge et al., 2019; Li et al., 2020a; Chen et al., 2022a). Finally, our results demonstrate the utility of contrastive representation learning for neuroimaging-based prediction of cognition. However, our proposed TractoSCR and permutation feature importance methods can be applied to other regression tasks.

## 5 Conclusion

In this work, we have proposed TractoSCR, a simple yet effective contrastive representation learning method for regression. We applied our TractoSCR method on multi-site harmonized dMRI tractography measures from the large-scale ABCD dataset (8,735 participants) to predict neurocognitive scores relating to general cognitive ability, executive function and learning/memory. We compared TractoSCR with several SOTA methods, and TractoSCR obtained significantly better prediction performance. Overall, we found that fiber clusters within the projection and superficial white matter were the most important for predicting neurocognitive scores.

## Data availability statement

The original contributions presented in the study are included in the article/Supplementary material, further inquiries can be directed to the corresponding authors.

## Ethics statement

The studies involving humans were approved by Brigham and Women's Hospital Institutional Review Board. The studies were conducted in accordance with the local legislation and institutional requirements. Written informed consent for participation in this study was provided by the participants' legal guardians/next of kin.

## Author contributions

TX: Conceptualization, Formal analysis, Methodology, Software, Validation, Visualization, Writing – original draft, Writing – review & editing. FZ: Conceptualization, Data curation, Methodology, Supervision, Validation, Writing – original draft, Writing – review & editing. LZ: Formal analysis, Investigation, Visualization, Writing – review & editing. CZ: Methodology, Supervision, Writing – review & editing. YC: Formal analysis, Investigation, Methodology, Writing – review & editing. SC-K: Data curation, Writing – review & editing. SP: Data curation, Software, Writing – review & editing. WW: Funding acquisition, Resources, Writing – review & editing. YR: Data curation, Funding acquisition, Resources, Writing – review & editing. NM: Data curation, Resources, Writing – review & editing. WC: Methodology, Resources, Supervision, Writing – review & editing, Project administration. LO'D: Conceptualization, Data curation, Funding acquisition, Investigation, Methodology, Resources, Supervision, Visualization, Writing – original draft, Writing – review & editing.

## References

[B1] Aracil-Bolaños I. Sampedro F. Marín-Lahoz J. Horta-Barba A. Martínez-Horta S. Botí M.. (2019). A divergent breakdown of neurocognitive networks in Parkinson's disease mild cognitive impairment. Hum. Brain Mapp. 40, 3233–3242. 10.1002/hbm.24593 30938027 PMC6865605

[B2] Arik S. Ö. Pfister T. (2021). TabNet: attentive interpretable tabular learning. AAAI 35, 6679–6687. 10.1609/aaai.v35i8.16826

[B3] Bahri D. Jiang H. Tay Y. Metzler D. (2022). SCARF: self-supervised contrastive learning using random feature corruption. arXiv:2106.15147. 10.48550/arXiv.2106.15147

[B4] Berger M. Pirpamer L. Hofer E. Ropele S. Duering M. Gesierich B.. (2022). Free water diffusion MRI and executive function with a speed component in healthy aging. Neuroimage 257:119303. 10.1016/j.neuroimage.2022.119303 35568345 PMC9465649

[B5] Borisov V. Leemann T. Sessler K. Haug J. Pawelczyk M. Kasneci G. (2021). Deep neural networks and tabular data: a survey. ArXiv. 10.48550/arXiv.2110.01889 37015381

[B6] Breiman L. (2001). Random forests. Mach. Learn. 45, 5–32. 10.1023/A:1010933404324

[B7] Brown S. S. G. Mak E. Clare I. Grigorova M. Beresford-Webb J. Walpert M.. (2022). Support vector machine learning and diffusion-derived structural networks predict amyloid quantity and cognition in adults with Down's syndrome. Neurobiol. Aging 115, 112–121. 10.1016/j.neurobiolaging.2022.02.013 35418341 PMC10327571

[B8] Casaletto K. B. Umlauf A. Beaumont J. Gershon R. Slotkin J. Akshoomoff N.. (2015). Demographically corrected normative standards for the English version of the NIH toolbox cognition battery. J. Int. Neuropsychol. Soc. 21, 378–391. 10.1017/S1355617715000351 26030001 PMC4490030

[B9] Casey B. J. Cannonier T. Conley M. I. Cohen A. O. Barch D. M. Heitzeg M. M.. (2018). The adolescent brain cognitive development (ABCD) study: imaging acquisition across 21 sites. Dev. Cogn. Neurosci. 32, 43–54. 10.1016/j.dcn.2018.03.001 29567376 PMC5999559

[B10] Cetin Karayumak S. Bouix S. Ning L. James A. Crow T. Shenton M.. (2019). Retrospective harmonization of multi-site diffusion MRI data acquired with different acquisition parameters. Neuroimage 184, 180–200. 10.1016/j.neuroimage.2018.08.073 30205206 PMC6230479

[B11] Cetin-Karayumak S. Zhang F. Billah T. Bouix S. Pieper S. O'Donnell L. J.. (2021). Harmonization of multi-site diffusion MRI data of the adolescent brain cognitive development (ABCD) study. in ISMRM, ed. J. H. Krystal (Amsterdam: Elsevier), 84.

[B12] Cetin-Karayumak S. Zhang F. O'Donnell L. J. Rathi Y. (2022). Harmonization of Multi-Site diffusion magnetic resonance imaging data from the adolescent brain cognitive development study. Biol. Psychiat. 91:S84. 10.1016/j.biopsych.2022.02.22723629049

[B13] Chamberland M. Genc S. Tax C. M. W. Shastin D. Koller K. Raven E. P.. (2021). Detecting microstructural deviations in individuals with deep diffusion MRI tractometry. Nat. Comput. Sci. 1, 598–606. 10.1038/s43588-021-00126-8 35865756 PMC7613101

[B14] Chen C. Yang X. Huang R. Hu X. Huang Y. Lu X.. (2022a). “Fine-Grained correlation loss for regression,” in MICCAI, eds. L. Wang, Q. Dou, P. T. Fletcher, S. Speidel, S. Li (Cham: Springer), 663–672.

[B15] Chen M. Li H. Fan H. Dillman J. R. Wang H. Altaye M.. (2022b). ConCeptCNN: a novel multi-filter convolutional neural network for the prediction of neurodevelopmental disorders using brain connectome. Med. Phys. 49, 3171–3184. 10.1002/mp.15545 35246986 PMC9164760

[B16] Chen M. Li H. Wang J. Yuan W. Altaye M. Parikh N. A.. (2020a). Early prediction of cognitive deficit in very preterm infants using brain structural connectome with transfer learning enhanced deep convolutional neural networks. Front. Neurosci. 14:858. 10.3389/fnins.2020.00858 33041749 PMC7530168

[B17] Chen T. Guestrin C. (2016). “XGBoost: a scalable tree boosting system,” in ACM SIGKDD (New York, NY: Association for Computing Machinery), 785–794.

[B18] Chen T. Kornblith S. Norouzi M. Hinton G. (2020b). “A simple framework for contrastive learning of visual representations,” in ICML, Vol. 119 (PMLR), 1597–1607.

[B19] Chen X. He K. (2021). “Exploring simple siamese representation learning,” in CVPR (New York City, NY: IEEE), 15750–15758

[B20] Chen Y. Zhang F. Zhang C. Xue T. Zekelman L. R. He J.. (2022c). “White matter tracts are point clouds: neuropsychological score prediction and critical region localization via geometric deep learning,” in MICCAI, eds. L. Wang, Q. Dou, P. T. Fletcher, S. Speidel, S. Li (Cham: Springer), 174–184.

[B21] Cui Z. Gong G. (2018). The effect of machine learning regression algorithms and sample size on individualized behavioral prediction with functional connectivity features. Neuroimage 178, 622–637. 10.1016/j.neuroimage.2018.06.001 29870817

[B22] Dai W. Li X. Chiu W. H. K. Kuo M. D. Cheng K.-T. (2022). Adaptive contrast for image regression in Computer-Aided disease assessment. IEEE Trans. Med. Imag. 41, 1255–1268. 10.1109/TMI.2021.3137854 34941504

[B23] Dubois J. Galdi P. Paul L. K. Adolphs R. (2018). A distributed brain network predicts general intelligence from resting-state human neuroimaging data. Philos. Trans. R. Soc. Lond. B Biol. Sci. 373:284. 10.1098/rstb.2017.0284 30104429 PMC6107566

[B24] Dufumier B. Gori P. Victor J. Grigis A. Wessa M. Brambilla P.. (2021). “Contrastive learning with continuous proxy meta-data for 3D MRI classification,” in MICCAI, eds. M. de Bruijne, P. C. Cattin, S. Cotin, N. Padoy, S. Speidel, Y. Zheng, C. Essert (Cham: Springer), 58–68.

[B25] Engilberge M. Chevallier L. Pérez P. Cord M. (2019). “SoDeep: a sorting deep net to learn ranking loss surrogates,” in CVPR (New York City, NY: IEEE), 10784–10793.

[B26] Feng G. Wang Y. Huang W. Chen H. Dai Z. Ma G.. (2022). Methodological evaluation of individual cognitive prediction based on the brain white matter structural connectome. Hum. Brain Mapp. 43, 3775–3791. 10.1002/hbm.25883 35475571 PMC9294303

[B27] Fisher C. K. Smith A. M. Walsh J. R. (2019). Machine learning for comprehensive forecasting of Alzheimer's disease progression. Sci. Rep. 9:13622. Available online at: https://www.nature.com/articles/s41598-019-49656-2 31541187 10.1038/s41598-019-49656-2 PMC6754403

[B28] Forkel S. J. Friedrich P. Thiebaut de Schotten M. Howells H. (2022). White matter variability, cognition, and disorders: a systematic review. Brain Struct. Funct. 227, 529–544. 10.1007/s00429-021-02382-w 34731328 PMC8844174

[B29] Goddings A.-L. Roalf D. Lebel C. Tamnes C. K. (2021). Development of white matter microstructure and executive functions during childhood and adolescence: a review of diffusion MRI studies. Dev. Cogn. Neurosci. 51:101008. 10.1016/j.dcn.2021.101008 34492631 PMC8424510

[B30] Gong W. Bai S. Zheng Y. Q. Smith S. M. Beckmann C. F. (2022). Supervised phenotype discovery from multimodal brain imaging. IEEE Trans. Med. Imaging. 2022:458926. 10.1101/2021.09.03.458926 PMC7618907 36318559

[B31] Gong W. Beckmann C. F. Smith S. M. (2021). Phenotype discovery from population brain imaging. Med. Image Anal. 71:102050. 10.1016/j.media.2021.102050 33905882 PMC8850869

[B32] Gorishniy Y. Rubachev I. Khrulkov V. Babenko A. (2021). “Revisiting deep learning models for tabular data,” in NeurIPS, eds. Ranzato, M. et al. (New Orleans: Neural Information Processing Systems Foundation, Inc.), 18932–18943.

[B33] Hastie T. Tibshirani R. Friedman J. (2009). “Ensemble learning,” in The Elements of Statistical Learning: Data Mining, Inference, and Prediction (Cham: Springer), 605–624.

[B34] He H. Zhang F. Pieper S. Makris N. Rathi Y. Wells W.. (2022). “Model and predict age and sex in healthy subjects using brain white matter features: a deep learning approach,” in ISBI (New York City, NY: IEEE), 1–5.

[B35] Jandric D. Parker G. J. M. Haroon H. Tomassini V. Muhlert N. Lipp I. (2022). A tractometry principal component analysis of white matter tract network structure and relationships with cognitive function in relapsing-remitting multiple sclerosis. Neuroimage Clin. 34:102995. 10.1016/j.nicl.2022.102995 35349892 PMC8958271

[B36] Jeong J. W. Lee M. H. O'Hara N. Juhász C. Asano E. (2021). Prediction of baseline expressive and receptive language function in children with focal epilepsy using diffusion tractography-based deep learning network. Epilepsy Behav. 117:107909. 10.1016/j.yebeh.2021.107909 33740493 PMC8035310

[B37] Jollans L. Boyle R. Artiges E. Banaschewski T. Desrivières S. Grigis A.. (2019). Quantifying performance of machine learning methods for neuroimaging data. Neuroimage 199, 351–365. 10.1016/j.neuroimage.2019.05.082 31173905 PMC6688909

[B38] Kadra A. Lindauer M. Hutter F. Grabocka J. (2021). “Well-tuned simple nets excel on tabular datasets,” in NeurISP, eds. Ranzato, M. et al. (New Orleans: Neural Information Processing Systems Foundation, Inc.), 23928–23941.

[B39] Khosla P. Teterwak P. Wang C. Sarna A. Tian Y. Isola P.. (2020). “Supervised contrastive learning,” in NeurIPS (New Orleans: Neural Information Processing Systems Foundation, Inc.), Vol. 33, 18661–18673.

[B40] Kim M. Bao J. Liu K. Park B. Y. Park H. Baik J. Y.. (2021). A structural enriched functional network: an application to predict brain cognitive performance. Med. Image Anal. 71:102026. 10.1016/j.media.2021.102026 33848962 PMC8184595

[B41] Lei W. Xu W. Gu R. Fu H. Zhang S. Zhang S.. (2021). “Contrastive learning of relative position regression for One-Shot object localization in 3D medical images,” in MICCAI, eds. M. de Bruijne, P. C. Cattin, S. Cotin, N. Padoy, S. Speidel, Y. Zheng, C. Essert (Cham: Springer), 155–165.

[B42] Li D. Jiang T. Jiang M. (2020a). “Norm-in-Norm loss with faster convergence and better performance for image quality assessment,” in ACM MM (New York, NY: Association for Computing Machinery), 789–797.

[B43] Li X. Wang Y. Wang W. Huang W. Chen K. Xu K.. (2020b). Age-Related decline in the topological efficiency of the brain structural connectome and cognitive aging. Cereb. Cortex 30, 4651–4661. 10.1093/cercor/bhaa066 32219315

[B44] Luciana M. Bjork J. M. Nagel B. J. Barch D. M. Gonzalez R. Nixon S. J.. (2018). Adolescent neurocognitive development and impacts of substance use: overview of the adolescent brain cognitive development (ABCD) baseline neurocognition battery. Dev. Cogn. Neurosci. 32, 67–79. 10.1016/j.dcn.2018.02.006 29525452 PMC6039970

[B45] Madole J. W. Ritchie S. J. Cox S. R. Buchanan C. R. Hernández M. V. Maniega S. M.. (2021). Aging-Sensitive networks within the human structural connectome are implicated in Late-Life cognitive declines. Biol. Psychiat. 89, 795–806. 10.1016/j.biopsych.2020.06.010 32828527 PMC7736316

[B46] Malcolm J. G. Shenton M. E. Rathi Y. (2010). Filtered multitensor tractography. IEEE Trans. Med. Imaging 29, 1664–1675. 10.1109/TMI.2010.2048121 20805043 PMC3045040

[B47] Mansour L. S Seguin C. Smith R. E. Zalesky A. (2022). Connectome spatial smoothing (CSS): concepts, methods, and evaluation. Neuroimage 250:118930. 10.1016/j.neuroimage.2022.118930 35077853

[B48] Mansour L. S. Tian Y. Yeo B. T. T. Cropley V. Zalesky A. (2021). High-resolution connectomic fingerprints: mapping neural identity and behavior. Neuroimage 229:117695. 10.1016/j.neuroimage.2020.117695 33422711

[B49] Merz E. C. Strack J. Hurtado H. Vainik U. Thomas M. Evans A.. (2022). Educational attainment polygenic scores, socioeconomic factors, and cortical structure in children and adolescents. Hum. Brain Mapp. 43, 4886–4900. 10.1002/hbm.26034 35894163 PMC9582364

[B50] Norton I. Essayed W. I. Zhang F. Pujol S. Yarmarkovich A. Golby A. J.. (2017). SlicerDMRI: open source diffusion MRI software for brain cancer research. Cancer Res. 77, e101–e103. 10.1158/0008-5472.CAN-17-0332 29092950 PMC5679308

[B51] Ooi L. Q. R. Chen J. Zhang S. Kong R. Tam A. Li J.. (2022). Comparison of individualized behavioral predictions across anatomical, diffusion and functional connectivity MRI. Neuroimage 263:119636. 10.1016/j.neuroimage.2022.119636 36116616

[B52] Pedregosa F. Varoquaux G. Gramfort A. Michel V. Thirion B. Grisel O.. (2011). Scikit-learn: machine learning in python. J. Mach. Learn. Res. 12, 2825–2830.

[B53] Pohl K. M. Thompson W. K. Adeli E. Linguraru M. G. (2019). “Adolescent brain cognitive development neurocognitive prediction,” in First Challenge, ABCD-NP 2019, Held in Conjunction with MICCAI (Cham: Springer).

[B54] Prokhorenkova L. Gusev G. Vorobev A. Dorogush A. V. Gulin A. (2018). “CatBoost: unbiased boosting with categorical features,” in NeurISP (New Orleans: Neural Information Processing Systems Foundation, Inc.), 6639–6649.

[B55] Radhakrishnan H. Bennett I. J. Stark C. E. (2022). Higher-order multi-shell diffusion measures complement tensor metrics and volume in gray matter when predicting age and cognition. Neuroimage 253:119063. 10.1016/j.neuroimage.2022.119063 35272021 PMC10538083

[B56] Reddy Raamana P. Strother C. S. (2017). Python class defining a machine learning dataset ensuring key-based correspondence and maintaining integrity. J. Open Source Softw. 2:382. 10.21105/joss.00382

[B57] Reddy C. P. Rathi Y. (2016). Joint Multi-Fiber NODDI parameter estimation and tractography using the unscented information filter. Front. Neurosci. 10:166. 10.3389/fnins.2016.00166 27147956 PMC4837399

[B58] Richie-Halford A. Yeatman J. D. Simon N. Rokem A. (2021). Multidimensional analysis and detection of informative features in human brain white matter. PLoS Comput. Biol. 17:e1009136. 10.1371/journal.pcbi.1009136 34181648 PMC8270416

[B59] Rumelhart D. E. Hinton G. E. Williams R. J. (1986). “Learning internal representations by error propagation,” in Parallel Distributed Processing: Explorations in the Microstructure of Cognition, Vol. 1: Foundations, eds. J. A. Anderson and E. Rosenfeld (Boston, MA: The MIT Press), 318–362.

[B60] Schiffer C. Amunts K. Harmeling S. Dickscheid T. (2021). “Contrastive representation learning for whole brain cytoarchitectonic mapping in histological human brain sections,” in ISBI (New York City, NY: IEEE), 603–606.

[B61] Seguin C. Tian Y. Zalesky A. (2020). Network communication models improve the behavioral and functional predictive utility of the human structural connectome. Netw. Neurosci. 4, 980–1006. 10.1162/netn_a_00161 33195945 PMC7655041

[B62] Seyfioğlu M. S. Liu Z. Kamath P. Gangolli S. Wang S. Grabowski T.. (2022). “Brain-Aware replacements for supervised contrastive learning in detection of Alzheimer's disease,” in MICCAI, eds. L. Wang, Q. Dou, P. T. Fletcher, S. Speidel, S. Li (Cham: Springer), 461–470. 10.1007/978-3-031-16431-6_44 PMC11056282 38680538

[B63] Sheng G. Wang Q. Pei C. Gao Q. (2022). Contrastive deep embedded clustering. Neurocomputing 514, 13–20.

[B64] Snyder H. R. Miyake A. Hankin B. L. (2015). Advancing understanding of executive function impairments and psychopathology: bridging the gap between clinical and cognitive approaches. Front. Psychol. 6:328. 10.3389/fpsyg.2015.00328 25859234 PMC4374537

[B65] Sripada C. Rutherford S. Angstadt M. Thompson W. K. Luciana M. Weigard A.. (2020). Prediction of neurocognition in youth from resting state fMRI. Mol. Psychiat. 25, 3413–3421. 10.1038/s41380-019-0481-6 31427753 PMC7055722

[B66] Sun L. Liang X. Duan D. Liu J. Chen Y. Wang X.. (2022). Structural insight into the individual variability architecture of the functional brain connectome. Neuroimage 259:119387. 10.1016/j.neuroimage.2022.119387 35752416

[B67] Thompson W. K. Barch D. M. Bjork J. M. Gonzalez R. Nagel B. J. Nixon S. J.. (2019). The structure of cognition in 9 and 10 year-old children and associations with problem behaviors: findings from the ABCD study's baseline neurocognitive battery. Dev. Cogn. Neurosci. 36:100606. 10.1016/j.dcn.2018.12.004 30595399 PMC6676481

[B68] Vaswani A. Shazeer N. Parmar N. Uszkoreit J. Jones L. Gomez A. N.. (2017). “Attention is all you need,” in NeurIPS (New Orleans: Neural Information Processing Systems Foundation, Inc.), 6000–6010.

[B69] Volkow N. D. Koob G. F. Croyle R. T. Bianchi D. W. Gordon J. A. Koroshetz W. J.. (2018). The conception of the ABCD study: from substance use to a broad NIH collaboration. Dev. Cogn. Neurosci. 32, 4–7. 10.1016/j.dcn.2017.10.002 29051027 PMC5893417

[B70] Wang Y. Metoki A. Alm K. H. Olson I. R. (2018). White matter pathways and social cognition. Neurosci. Biobehav. Rev. 90, 350–370. 10.1016/j.neubiorev.2018.04.015 29684403 PMC5993647

[B71] Weerasekera A. Ion-Mărgineanu A. Green C. Mody M. Nolan G. P. (2023). Predictive models demonstrate age-dependent association of subcortical volumes and cognitive measures. Hum. Brain Mapp. 44, 801–812. 10.1002/hbm.26100 36222055 PMC9842902

[B72] Wu J. Li J. Eickhoff S. B. Hoffstaedter F. Hanke M. Yeo B. T. T.. (2022). Cross-cohort replicability and generalizability of connectivity-based psychometric prediction patterns. Neuroimage 262:119569. 10.1016/j.neuroimage.2022.119569 35985618 PMC9611632

[B73] Xue T. Zhang F. Zhang C. Chen Y. Song Y. Golby A. J.. (2023). Superficial white matter analysis: an efficient point-cloud-based deep learning framework with supervised contrastive learning for consistent tractography parcellation across populations and dMRI acquisitions. Med. Image Anal. 85:102759. 10.1016/j.media.2023.102759 36706638 PMC9975054

[B74] Xue T. Zhang F. Zhang C. Chen Y. Song Y. Makris N.. (2022). “SupWMA: consistent and efficient tractography parcellation of superficial white matter with deep learning,” in ISBI, eds. N. Ayache and J. Duncan (Amsterdam: Elsevier), 1–5.

[B75] Yoon J. Zhang Y. Jordon J. van der Schaar M. (2020). VIME: extending the success of self- and semi-supervised learning to tabular domain. NeurIPS 33, 11033–11043.

[B76] Zekelman L. R. Zhang F. Makris N. He J. Chen Y. Xue T.. (2022). White matter association tracts underlying language and theory of mind: an investigation of 809 brains from the human connectome project. Neuroimage 246:118739. 10.1016/j.neuroimage.2021.118739 34856375 PMC8862285

[B77] Zhang F. Daducci A. He Y. Schiavi S. Seguin C. Smith R. E.. (2022a). Quantitative mapping of the brain's structural connectivity using diffusion MRI tractography: a review. Neuroimage 249:118870. 10.1016/j.neuroimage.2021.118870 34979249 PMC9257891

[B78] Zhang F. Karayumak S. C. Pieper S. O'Donnell L. J. (2022b). “Consistent white matter parcellation in adolescent brain cognitive development (ABCD): a 10 k harmonized,” in ISMRM.

[B79] Zhang F. Noh T. Juvekar P. Frisken S. F. Rigolo L. Norton I.. (2020). SlicerDMRI: diffusion MRI and tractography research software for brain cancer surgery planning and visualization. JCO Clin. Cancer Inform. 4, 299–309. 10.1200/CCI.19.00141 32216636 PMC7113081

[B80] Zhang F. Wu Y. Norton I. Rathi Y. Golby A. J. O'Donnell L. J. (2019). Test-retest reproducibility of white matter parcellation using diffusion MRI tractography fiber clustering. Hum. Brain Mapp. 40, 3041–3057. 10.1002/hbm.24579 30875144 PMC6548665

[B81] Zhang F. Wu Y. Norton I. Rigolo L. Rathi Y. Makris N.. (2018). An anatomically curated fiber clustering white matter atlas for consistent white matter tract parcellation across the lifespan. Neuroimage 179, 429–447. 10.1016/j.neuroimage.2018.06.027 29920375 PMC6080311

[B82] Zhang Y. Li M. Ji Z. Fan W. Yuan S. Liu Q.. (2021). Twin self-supervision based semi-supervised learning (TS-SSL): retinal anomaly classification in sd-oct images. Neurocomputing 462, 491–505. 10.1016/j.neucom.2021.08.051

[B83] Zou H. Hastie T. (2005). Regularization and variable selection via the elastic net. J. R. Stat. Soc. Series B Stat. Methodol. 67, 301–320. 10.1111/j.1467-9868.2005.00503.x

