# Supplementary material for: TractoSCR: a novel supervised contrastive regression framework for prediction of neurocognitive measures using multi-site harmonized diffusion MRI tractography
Source: Front Neurosci. 2024 Jun 26;18:1411797. doi: 10.3389/fnins.2024.1411797 (PMC11233814; doi:10.3389/fnins.2024.1411797)
Supplement: Supplementary file 1 [file Data_Sheet_1.PDF]

TractoSCR: A Novel Supervised Contrastive Regression Framework for Prediction of Neurocognitive Measures Using Multi-Site Harmonized Diffusion MRI Tractography

Tengfei Xue[1,2], Fan Zhang[1,3*], Leo R. Zekelman[1], Chaoyi Zhang[2], Yuqian Chen[1], Suheyla Cetin-Karayumak[1], Steve Pieper[1], William M. Wells[1], Yogesh Rathi[1], Nikos Makris[1], Weidong Cai[2], and Lauren J. O'Donnell[1,*]
[1]Brigham and Women's Hospital, Harvard Medical School, Boston, USA
[2]School of Computer Science, University of Sydney, Sydney, Australia
[3]School of Information and Communication Engineering, University of Electronic Science and Technology of China, Chengdu, China

Supplementary Table S1. Indices and tract locations of the top 50 important clusters for PC1, PC2 and PC3 predictions. The tract names and categories are listed as in the ORG atlas (Zhang et al., 2018). Hemispheric localization of each cluster is provided in the cluster index (L_, R_, C_ for left, right, or commissural/decussating, respectively). Tracts in bold text have clusters that are important across all the three prediction tasks.

Tract information			PC1 - General Ability		PC2 - Executive Function		PC3 - Learning/Memory		Total Number of Predictive Clusters
Category	Tract Name	Total Clusters in Atlas	Number of Predictive Clusters	Cluster Indices	Number of Predictive Clusters	Cluster Indices	Number of Predictive Clusters	Cluster Indices	
**Association tracts**	arcuate fasciculus (AF)	11	0		2 (18.18%)	L_169, R_169	0		2
	**cingulum bundle (CB)**	22	2 (9.09%)	L_141, L_682	4 (18.18%)	L_682, R_141, R_682, R_549	2 (9.09%)	L_46, R_313	8
	external capsule (EC)	4	0		0		1 (25.00%)	R_721	1
	extreme capsule (EmC)	3	0		0		2 (66.67%)	L_776, L_778	2
	inferior longitudinal fasciculus (ILF)	15	1 (6.67%)	R_167	0		1 (6.67%)	L_71	2
	inferior occipito-frontal fasciculus	8	1 (12.50%)	L_716	0		3 (37.50%)	L_681, L_716, L_752	4

Association tracts	(IOFF)								
	**middle longitudinal fasciculus (MdLF)**	15	2 (13.33%)	R_48, R_472	1 (6.67%)	L_48	4 (26.67%)	R_472, R_48, L_48, L_559	7
	superior longitudinal fasciculus I (SLF-I)	18	0		0		0		0
	superior longitudinal fasciculus II (SLF-II)	12	2 (16.67%)	R_178, R_21	0		0		2
	superior longitudinal fasciculus III (SLF-III)	5	0		0		0		0
	uncinate fasciculus (UF)	7	0		0		0		0
Projection tracts	corticospinal tract (CST)	11	0		0		0		0
	corona-radiata-frontal (excluding the CST) (CR-F)	8	2 (25.00%)	R_748, R_75	1 (12.50%)	L_675	0		3
	**corona-radiata-parietal (excluding the CST) (CR-P)**	2	1 (50.00%)	L_137	1 (50.00%)	R_148	1 (50.00%)	L_137	3
	**striato-frontal (SF)**	15	5 (33.33%)	R_237, L_237, R_328, R_586, L_223	2 (13.33%)	L_237, R_237	3 (20.00%)	L_237, R_223, R_31	10
	striato-occipital (SO)	1	1 (100.00%)	R_564	0		0		1
	striato-parietal (SP)	2	1 (50.00%)	R_347	0		1 (50.00%)	R_347	2

**Projection tracts**	**thalamo-frontal (TF)**	21	1 (4.76%)	R_64	1 (4.76%)	L_735	7 (33.33%)	L_224, L_196, L_273, L_688, L_19, R_64, L_735	9
	thalamo-occipital (TO)	3	1 (33.33%)	R_534	0		0		1
	**thalamo-temporal (TT)**	9	1 (11.11%)	R_732	2 (22.22%)	R_784, R_794	1 (11.11%)	L_732	4
	**thalamo-parietal (TP)**	10	1 (10.00%)	R_6	1 (10.00%)	L_499	1 (10.00%)	L_475	3
	posterior limb of internal capsule (PLIC)	2	1 (50.00%)	L_318	1 (50.00%)	L_318	0		2
**Commissural tracts**	corpus callosum 1 (CC1)	3	2 (66.67%)	C_654, C_634	0		0		2
	**corpus callosum 2 (CC2)**	16	4 (25.00%)	C_664, C_588, C_372, C_628	1 (6.25%)	C_598	1 (6.25%)	C_364	6
	corpus callosum 3 (CC3)	8	0		0		0		0
	corpus callosum 4 (CC4)	6	0		0		1 (16.67%)	C_351	1
	corpus callosum 5 (CC5)	5	0		0		0		0
	**corpus callosum 6 (CC6)**	14	1 (7.14%)	C_457	1 (7.14%)	C_457	1 (7.14%)	C_486	3
	corpus callosum 7 (CC7)	7	3 (42.86%)	C_92, C_7, C_4	0		0		3
**Cerebellar tracts**	cortico-ponto-cerebellar (CPC)	5	0		1 (20.00%)	C_678	0		1
	inferior	3	1 (33.33%)	R_515	0		3	R_126,	4

**Cerebellar tracts**	cerebellar peduncle (ICP)						(100.00%)	L_126, R_13	
	intracerebellar input and Purkinje tract (Intra-CBLM-I-P)	14	2 (14.29%)	R_19, L_54	1 (7.14%)	L_19	0		3
	**intracerebellar parallel tract (Intra-CBLM-PaT)**	25	2 (8.00%)	L_512, L_513	2 (8.00%)	L_529, R_539	3 (12.00%)	R_539, L_495, L_539	7
	middle cerebellar peduncle (MCP)	9	0		1 (11.11%)	C_115	0		1
**Superficial tracts**	**superficial-frontal (Sup-F)**	81	6 (7.41%)	R_346, R_293, R_299, R_388, R_262, L_629	11 (13.58%)	L_637, L_47, L_262, L_585, L_338, R_262, R_35, R_585, L_346, L_3, L_619	3 (3.70%)	R_619, R_657, R_656	20
	superficial-frontal-parietal (Sup-FP)	10	0		2 (20.00%)	L_479, R_477	2 (20.00%)	R_391, R_398	4
	superficial-occipital (Sup-O)	7	0		2 (28.57%)	R_78, L_83	0		2
	superficial-occipital-temporal (Sup-OT)	7	0		5 (71.43%)	L_95, L_569, R_554, R_95, R_553	3 (42.86%)	R_553, L_554, L_553	8
	**superficial-parietal (Sup-P)**	40	4 (10.00%)	L_439, L_421, L_392, L_46	2 (5.00%)	R_18, R_23	3 (7.50%)	R_439, R_274, L_439	9
	superficial-parietal-occipital (Sup-PO)	11	1 (9.09%)	R_75	1 (9.09%)	L_84	0		2
	superficial-pari	24	1 (4.17%)	R_12	1 (4.17%)	L_82	0		2

**Superficial tracts**	etal-temporal (Sup-PT)								
	superficial-temporal (Sup-T)	14	0		3 (21.43%)	R_556, L_155, R_74	3 (21.43%)	R_729, L_729, R_795	6

F. Zhang *et al.*, “An anatomically curated fiber clustering white matter atlas for consistent white matter tract parcellation across the lifespan,” *Neuroimage*, vol. 179, pp. 429–447, Oct. 2018.
